# A g-local call for culturally responsive prevention and care of gender-based violence against women migrants and refugees

**DOI:** 10.3389/fsoc.2025.1651018

**Published:** 2025-09-30

**Authors:** Mohamed Taiebine

**Affiliations:** Euromed Research Center, Euro-Mediterranean University of Fez (UEMF), Fez, Morocco

**Keywords:** women, migrants, wellbeing, g-local, prevention, cultural, gender-based violence (GBV)

## Introduction

Morocco's migration context encompasses diverse forms including transit, settlement, and return by individuals from various nationalities, mainly from sub-Saharan Africa (SSA; Keygnaert et al., 2014). This complexity affects efforts to address gender-based violence (GBV), as migrants' vulnerabilities vary significantly. Structural violence through systemic inequalities perpetuates GBV by making migrant women vulnerable.

Broadly speaking, GBV includes intimate partner violence (IPV), child abuse, female genital mutilation/cutting, child marriage, and non-partner sexual violence (Decker et al., 2015; Grose et al., 2021). It manifests as physical, sexual, emotional, and structural violence, with emerging concerns about technology-facilitated GBV (Bansal et al., 2023). The prevalence varies between high-income countries (HICs) and low- and middle-income countries (LMICs), with lifetime physical or sexual IPV among adolescent women in LMICs at 28% and young adult women at 29%, highest in East and Southern Africa (Decker et al., 2015).

Generally speaking, GBV in Morocco shares similarities with other LMICs regarding effects on women and patriarchal norms but has distinct features shaped by its socio-cultural environment. Morocco's role as a transit and destination hub for migrants adds complexity to GBV issues. According to the Haut-Commissariat au Plan (Haut-Commissariat au Plan, 2022), 57.1% of Moroccan women aged 15 to 74 experienced physical, psychological, sexual, or economic violence in the preceding 12 months.

In recent years, Morocco has become a destination for migrants, particularly from sub-Saharan Africa (SSA). The country faces challenges in managing migration flows and integrating newcomers (Merrouni and Machak, 2019; Nova et al., 2019). Morocco attracted 102,400 migrants, with nearly half in irregular situations, creating challenges for both migrants and the host country (Norman and Reiling, 2024). While Morocco shows commitment to addressing GBV through legal frameworks and national strategies, challenges persist, including legal loopholes and limited protection against non-physical abuse. Programs face sustainability issues due to heavy reliance on donor funding (International Organization for Migration, 2023; Organization for Economic Co-operation and Development, 2024).

This viewpoint article addresses female migrants and refugees' mental and neurocognitive health challenges related to GBV in Morocco, based on a qualitative synthesis of literature, policy documents, and expert insights, including peer-reviewed articles and legal texts. Although the recommendations provided, such as eco-systemic strategies, culturally sensitive care, and community-driven initiatives (Sisic et al., 2024; Holtmann et al., 2023), are not entirely new, the objective is to consolidate these well-established concepts, accommodate them, and emphasize their critical relevance within Morocco's migration context.

## Discussion

### G-local regulations toward GBV in women migrants and refugees

In public health, “g-local” describes the relationship between global and local elements shaping health outcomes. While health issues manifest locally, they are influenced by global trends, policies, and standards, with successful local initiatives informing global strategies (Rowthorn, 2015).

Moving to a broader perspective, The implementation of global-local regulations for sustainable development in Morocco shows mixed outcomes in economic growth and healthcare (Ziky and El-Abdellaoui, 2023). This affects the country's ability to train and retain healthcare professionals, crucial for achieving Sustainable Development Goal 3 (Elomrani et al., 2024; Taiebine, 2025).

On the one hand, The International Organization for Migration (IOM) operates within Morocco's 2013 National Strategy on Migration and Asylum, promoting rights-based migration management. IOM's work aligns with the National Strategic Plan on Migration Health (2021–2025) and health reforms, including Laws 09–21 and 06–22, which aim to improve healthcare access for all residents (International Organization for Migration, 2023).

On the other hand, looking at the broader global context, The Third Global Consultation on the Health of Refugees and Migrants (World Health Organization, 2024) emphasized country-led initiatives and equitable healthcare access regardless of documentation status (World Health Organization, 2024).

Moreover, the Moroccan government has shown its dedication to combating GBV by enacting Law 103.13 in 2018, which criminalizes forced marriages and provides a clear definition of sexual harassment (International Organization for Migration, 2023; World Health Organization, 2024). There have also been significant efforts to train professionals, such as police officers, social workers, and healthcare providers, who assist survivors of violence against women, often in partnership with international organizations like UN Women and United Nations sexual and reproductive health agency (UNFPA).

While legal frameworks represent a crucial step in mitigating GBV, they often do not fully account for its profound individual consequences. GBV's impact extends beyond legal definitions into mental, psychosocial, and neuropsychological health. This section shifts from policy to individual level, exploring how violence affects cognitive and emotional wellbeing of survivors, particularly within vulnerable migrant populations.

### The mental, psychosocial and neuropsychological consequences of GBV

Numerous studies have examined the relationship between GBV and neurodevelopmental vulnerability among African migrant women in Morocco (Mekki-Berrada, 2019). Conditions like autism, developmental delays, and intellectual disabilities increase GBV likelihood (Nouar et al., 2024). These vulnerabilities stem from communication challenges, social isolation, and limited understanding of personal safety (Graham et al., 2016). Premorbid trauma and adverse childhood experiences can increase susceptibility to exploitation.

For migrant and refugee women in Morocco, early detection and intervention are crucial for addressing mental health concerns (Schmengler et al., 2019, 2021; Taiebine, 2025). GBV-related trauma, displacement, and isolation can lead to depression, anxiety, PTSD, and cognitive impairments (Essayagh et al., 2023a,b; James et al., 2022). Standard assessment tools often fail to capture trauma-related factors, while cultural and sociolinguistic barriers (Mezzatesta Gava et al., 2022) impede effective evaluation.

### G-local policy, integrated care pathways and clinical recommendations

When examining GBV against women migrants and refugees, it becomes evident that international frameworks concerning human rights, refugee protection, and violence against women set forth global standards and legal responsibilities. These international standars subsequently impact national policies and the obligations of host nations, such as Morocco, in delivering protection and services. Nevertheless, the lived experiences of GBV, its frequency, the various forms it assumes, and the obstacles to obtaining support are profoundly influenced by local socio-cultural contexts, social dynamics, and the unique vulnerabilities encountered by migrant and refugee women in those environments (International Organization for Migration, 2023).

In this context, Morocco has taken significant measures to support GBV survivors, including the implementation of provisions in its Action Plan (2021–2024), which led to the establishment of partnership agreements with 44 associations and the creation of 82 safe spaces to accommodate abused women seeking protection. These efforts are complemented by diversified hybrid platforms for reporting violence, utilizing a vast network of hotlines and mailing addresses to ensure accessibility for all women, including migrants (International Organization for Migration, 2023).

While Morocco has made strides with laws like Law 103.13, which criminalizes some forms of violence, critical challenges in practical implementation remain. Migrant women, especially those in irregular situations, face significant barriers. These include language barriers, a lack of legal awareness, and a powerful fear of deportation that deters them from reporting abuse. Such systemic obstacles highlight that legal frameworks, while important, must be complemented by effective, on-the-ground support and a deeper understanding of the unique vulnerabilities of migrant women to be truly effective.

### Toward an integrated psycho-social care and wellbeing

**Figure 1:**
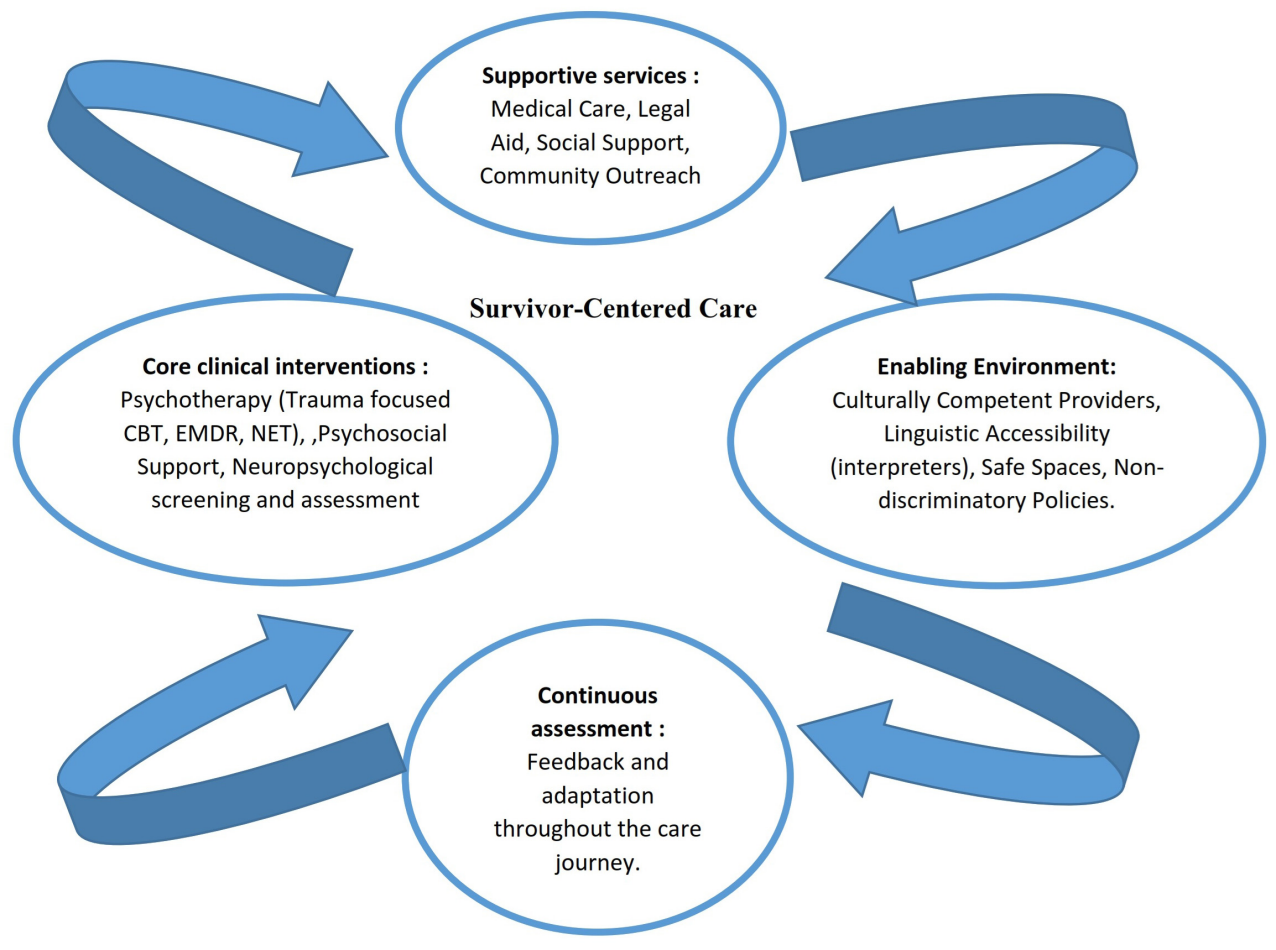
Suggested framework for an integrated psycho-social clinical pathway care for migrants GBV survivors.

Given the polymorphic challenges faced by women migrants and refugees, an integrated psycho-social clinical pathway care is needed (Figure 1), using an eco-systemic and bio-ecological approach to address mental health issues (World Health Organization, 2018). The eco-systemic framework emphasizes the overlap between individuals and their environments, while the bio-ecological model highlights the nested structures of these environments and their interactions over time (Sisic et al., 2024; Holtmann et al., 2023).

The eco-systemic model shows that a migrant woman's vulnerability to GBV is influenced by multiple levels: microsystem (immediate family), mesosystem (interactions between home and social networks), exosystem (community services), and macrosystem (societal laws and norms). Alternatively, the bio-ecological model adds the chronosystem, accounting for time and life transitions in migrant women's GBV experiences, including pre-migration trauma and adaptation. A salutogenic approach focuses on enhancing resilience and wellbeing by strengthening a woman's Sense of Coherence (SOC)—her ability to comprehend, manage, and find meaning in challenges.

### Trauma-informed assessment and care

Consequently, alongside this holistic perspective as illustrated in Figure 1, it is also crucial to improve access to mental health services delivered by professionals who are culturally competent and sensitive to the unique experiences and needs of these women (Taiebine, 2025). Moreover, recognizing the significant role of the social environment, community-based initiatives (Grose et al., 2019) that provide support, education, and resources should be established. In tandem with these efforts, it is essential to address social determinants of health, such as poverty, discrimination, and inadequate social support, as these factors can significantly affect the mental health of migrant and refugee women (Barakat et al., 2022; Rabbani et al., 2024).

Finally, acknowledging the high prevalence of trauma in this population, there is an urgent requirement for trauma-informed therapeutic approaches. These include Narrative Exposure Therapy (NET), a short-term, structured approach for single and multiple traumas; Trauma-Focused Cognitive Behavioral Therapy (TF-CBT), a psychosocial treatment model designed to treat post-traumatic stress and other trauma-related issues in children, adolescents, and adults; and Eye Movement Desensitization and Reprocessing (EMDR), a psychotherapy treatment designed to alleviate the distress associated with traumatic memories. These psychological interventions and mental health strategies should be specifically designed for women migrants and refugees in Morocco (Rabbani et al., 2024).

To tackle GBV against women effectively, Morocco needs stronger legal frameworks, coordination, and sustainable funding. Challenges like under-reporting, limited shelters, and social stigma necessitate victim-centered services and awareness to encourage reporting (Ennaji, 2011; Wafqui et al., 2024).

Evidence-based resources in multiple languages are needed to address mental and cognitive challenges of GBV among women migrants and refugees in Morocco (Acharai et al., 2023). While initiatives like hotline 8,350, “Kolona Maak” platform and Najatbot AI provide support, their effectiveness is limited by internet access and digital literacy requirements. The platform's ability to navigate legal barriers for irregular migrants remains unclear (Benachir and Raïs, 2023; Mrabti and Alaoui, 2025). Safe spaces, though crucial, are mainly urban-centered and often inaccessible to irregular migrants due to deportation fears and distrust in official systems. External funding dependency raises sustainability concerns.

### Cross-culturally adapted neuropsychological interventions

To effectively conduct neuropsychological screening and assessments for women migrants and refugees who have faced GBV at various stages—acute, sub-acute, and chronic—a trauma-informed and driven framework is vital (Santos Aurélio et al., 2022). The acute stage requires safety focus, with brief cognitive screening when appropriate. Sub-acute phase demands rapport building and trigger-free environments, using culturally sensitive tools and trained interpreters. Chronic stage assessment must consider PTSD-related cognitive challenges, allowing breaks and emotional support. All phases require monitoring emotional readiness and interpreting results within trauma and cultural contexts.

Developing culturally appropriate assessments requires considering linguistic subtleties, cultural values, and experiences of women migrants who are victims of GBV. This involves qualitative research to identify culturally significant constructs and modify assessment tools for language biases, interpretation, and response tendencies. Validation studies using psychometric techniques (Nguyen et al., 2024), including linguistic validation, content validation by cultural specialists, and construct validation, are essential for reliability in the Moroccan context.

Future research should integrate mobile health solutions and conduct longitudinal studies to monitor women's wellbeing across migration phases, providing insights into long-term effects of displacement and trauma. This approach enables understanding how individual, familial, and societal elements influence female migrants' experiences, aligning with ecosystemic frameworks across multiple levels.

## Conclusion

Addressing GBV against migrant women in Morocco requires a coordinated g-local approach to address mental health needs. However, legal frameworks' effectiveness is limited by deportation fears, lack of awareness, and language barriers. Therefore, this article advocates for an integrated neuro-psycho-social care model through community-based initiatives and trauma-informed interventions.

Morocco has improved women migrants' welfare through access to services and protective measures. To advance these achievements, efforts must integrate initiatives within Morocco's sociocultural context as a host and transit country. Developing personalized, legally compliant services, alongside culturally appropriate assessments and interventions, may enhance inclusion and sustainable development.
